# Rotavirus Stimulates Release of Serotonin (5-HT) from Human Enterochromaffin Cells and Activates Brain Structures Involved in Nausea and Vomiting

**DOI:** 10.1371/journal.ppat.1002115

**Published:** 2011-07-14

**Authors:** Marie Hagbom, Claudia Istrate, David Engblom, Thommie Karlsson, Jesus Rodriguez-Diaz, Javier Buesa, John A. Taylor, Vesa-Matti Loitto, Karl-Eric Magnusson, Håkan Ahlman, Ove Lundgren, Lennart Svensson

**Affiliations:** 1 Division of Molecular Virology, Medical Faculty, University of Linköping, Linköping, Sweden; 2 Unidade de Biologia Molecular, Centro de Malaria e Outras Doenças Tropicais, Instituto de Higiene e Medicina Tropical, Universidade Nova de Lisboa, Lisboa, Portugal; 3 Division of Cell Biology, Medical Faculty, University of Linköping, Linköping, Sweden; 4 Division of Medical Microbiology, Medical Faculty, University of Linköping, Linköping, Sweden; 5 Department of Microbiology, School of Medicine, University of Valencia, Valencia, Spain; 6 School of Biological Sciences, University of Auckland, Auckland, New Zealand; 7 Department of Surgery, University of Gothenburg, Gothenburg, Sweden; 8 Department of Physiology, University of Gothenburg, Gothenburg, Sweden; Baylor College of Medicine, United States of America

## Abstract

Rotavirus (RV) is the major cause of severe gastroenteritis in young children. A virus-encoded enterotoxin, NSP4 is proposed to play a major role in causing RV diarrhoea but how RV can induce emesis, a hallmark of the illness, remains unresolved. In this study we have addressed the hypothesis that RV-induced secretion of serotonin (5-hydroxytryptamine, 5-HT) by enterochromaffin (EC) cells plays a key role in the emetic reflex during RV infection resulting in activation of vagal afferent nerves connected to nucleus of the solitary tract (NTS) and area postrema in the brain stem, structures associated with nausea and vomiting. Our experiments revealed that RV can infect and replicate in human EC tumor cells *ex vivo* and *in vitro* and are localized to both EC cells and infected enterocytes in the close vicinity of EC cells in the jejunum of infected mice. Purified NSP4, but not purified virus particles, evoked release of 5-HT within 60 minutes and increased the intracellular Ca^2+^ concentration in a human midgut carcinoid EC cell line (GOT1) and *ex vivo* in human primary carcinoid EC cells concomitant with the release of 5-HT. Furthermore, NSP4 stimulated a modest production of inositol 1,4,5-triphosphate (IP_3_), but not of cAMP. RV infection in mice induced Fos expression in the NTS, as seen in animals which vomit after administration of chemotherapeutic drugs. The demonstration that RV can stimulate EC cells leads us to propose that RV disease includes participation of 5-HT, EC cells, the enteric nervous system and activation of vagal afferent nerves to brain structures associated with nausea and vomiting. This hypothesis is supported by treating vomiting in children with acute gastroenteritis with 5-HT_3_ receptor antagonists.

## Author Summary

Rotavirus (RV) can cause severe dehydration and is a leading cause of childhood deaths worldwide. While most deaths occur due to excessive loss of fluids and electrolytes through vomiting and diarrhoea, the pathophysiological mechanisms that underlie this life-threatening disease remain to be clarified. Our previous studies revealed that drugs that inhibit the function of the enteric nervous system can reduce symptoms of RV disease in mice. In this study we have addressed the hypothesis that RV infection triggers the release of serotonin (5-hydroxytryptamine, 5-HT) from enterochromaffin (EC) cells in the intestine leading to activation of vagal afferent nerves connected to brain stem structures associated with vomiting. RV activated Fos expression in the nucleus of the solitary tract of CNS, the main target for incoming fibers from the vagal nerve. Both secreted and recombinant forms of the viral enterotoxin (NSP4), increased intracellular Ca^2+^ concentration and released 5-HT from EC cells. 5-HT induced diarrhoea in mice within 60 min, thereby supporting the role of 5-HT in RV disease. Our study provides novel insight into the complex interaction between RV, EC cells, 5-HT and nerves.

## Introduction

Rotavirus (RV) is the major cause of infantile gastroenteritis worldwide and the infection is associated with approximately 600,000 deaths every year, predominantly in developing countries [1]. Most of the deaths result from excessive loss of fluids and electrolytes through vomiting and diarrhoea. Despite its significant clinical importance and the research conducted over several decades, knowledge of the pathophysiological mechanisms that underpin this life-threatening disease remains limited. Several mechanisms have been proposed to account for the watery diarrhoea associated with RV infection. These include osmotic diarrhoea following a virus-induced loss of epithelial absorptive function, the effect of NSP4, a virus-encoded enterotoxin, and an active role of the enteric nervous system (ENS) and serotonin (5-hydroxytryptamine, 5-HT) [2]–[5]. However, the pathophysiological basis of virus-induced emesis, a hallmark of illnesses caused by RV and norovirus, is poorly understood.

The human ENS contains about 100 million neurones which are sensory-, inter- and motor neurons [6]. The luminal enterochromaffin (EC) cells “taste” and “sense” the luminal contents and can release mediators such as 5-HT to activate ENS as well as extrinsic vagal afferents to the brain. 5-HT is located in the secretory granules of the EC cells, which are most abundant in the duodenum and comprise the single largest enteroendocrine cell population. They are strategically positioned in the intestinal mucosa to release mediators of endocrine signalling from the basolateral surface to activate afferent neuron endings within the *lamina propria* [7], [8]. Following stimulation by several agents (e.g. hyperosmolarity, carbohydrates, mechanical distortion of the mucosa, cytostatic drugs) including the cholera toxin [8], [9], EC cells mobilize intracellular Ca^2+^ and release 5-HT [10]. 5-HT is involved in the regulation of gut motility, intestinal secretion, blood flow and several gastrointestinal disorders [11]–[15] including RV diarrhoea [3], chemotherapy-induced nausea and vomiting [16], [17] and *Staphylococcal* enterotoxin-induced vomiting [18].

We have previously shown that RV infection results in stimulation of the ENS and that RV diarrhoea in mice can be attenuated with 5-HT_3_ receptor antagonists, such as granisetron [3]. This drug is frequently used to reduce vomiting in humans [19], including children with acute gastroenteritis [20]–[23], suggesting that EC cells could be an important mediator of RV-induced diarrhoea and vomiting, symptoms regarded as naturally inherited host responses [24]. The proposed anti-emetic mechanism of 5-HT_3_ receptor blockade involves an action on receptors located on vagal afferents communicating with the medullary vomiting centre [25], [26] which is supported by observations that the stimulation of vagal 5-HT_3_ receptors in ferrets, shrews and dogs triggers an emetic reflex, evoking reverse peristalsis in response to chemotherapeutic agents and radiation treatment [19], [27]–[29].

The present study was designed to test the novel hypothesis that RV can stimulate human EC cells in the gut, causing release of 5-HT, which activates vagal afferents and the brain stem vomiting centre, a reaction cascade associated with vomiting.

## Results

### Rotavirus infects and replicates *ex vivo* and *in vitro* in human enterochromaffin tumor cells

To determine whether RV can infect EC cells, primary EC tumor cells (t.c.) were harvested from patients with liver metastasis undergoing surgery for the midgut carcinoid syndrome. In addition, a human midgut carcinoid t.c. line (GOT1), was examined for susceptibility to RV infection. The EC cell phenotype was confirmed by staining for 5-HT and the neuroendocrine granule marker chromogranin A. Immunofluorescence microscopy revealed that 100% of the GOT1 cells and 95% of the primary EC t.c. were positive for chromogranin A, and 68% of GOT1 cells and 40% of the primary EC t.c. were positive for 5-HT (Figure. S1); this confirmed that the vast majority of the cells were EC specific. To determine whether RV replicates in EC cells, we infected confluent GOT1 cells (200,000 cells/well) with trypsin-activated Rhesus rotavirus (RRV) (MOI 0,1), collected cells and media at 3, 24 and 48 h post infection (p.i.) and determined the progeny viral titre. The EC t.c. did indeed support RRV replication (Figure. S2) and the viral titres increased >100 fold from ≤1×10^1^ plaque forming units (pfu)/ml in samples collected 3 h p.i. to 2×10^3^ pfu/ml at 48 h p.i.

### Rotavirus infection increases the intracellular Ca^2+^ concentration and stimulates 5-HT release from human enterochromaffin tumor cells

RV infection has previously been shown to increases the intracellular Ca^2+^ concentration in human intestinal cells [30]–[34]. To investigate whether RRV has a similar effect on GOT1 and primary t.c., the cells were loaded with the ratiometric fluorescent Ca^2+^ indicator Fura-2 prior to infection with trypsin-activated RRV at an MOI of 10 and 3 respectively (Protocol. S1). The Ca^2+^-images were captured at 1, 5 and 23 h p.i. These experiments showed that RV infection caused an elevation in intracellular Ca^2+^ in EC t.c. within 60 min. (Protocol. S2, Figure. S3). GOT1 cells had a maximum release of Ca^2+^ at 60 min p.i. and primary cells had a maximum release at 5 h p.i.

**Figure 1:**
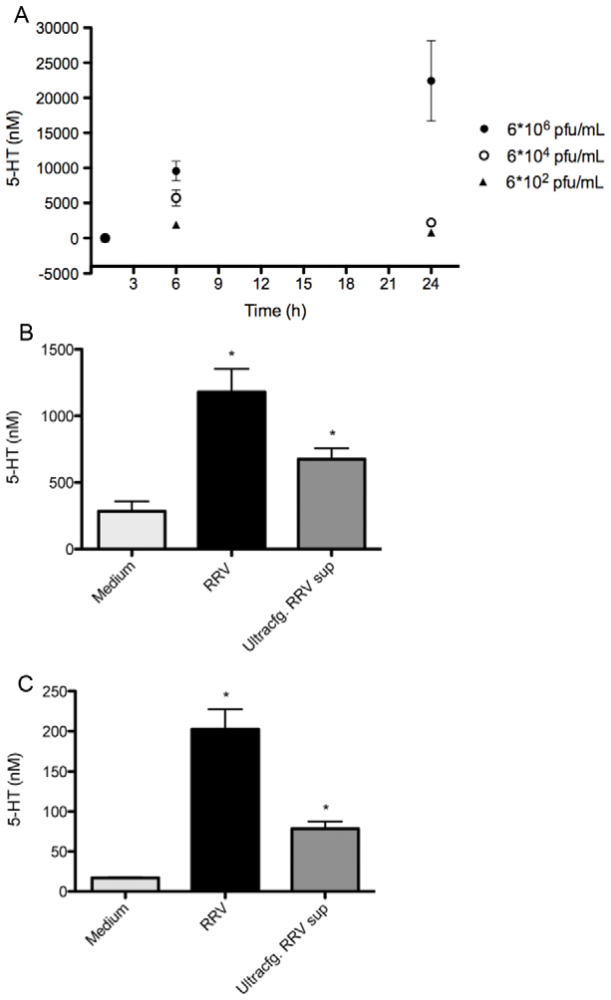
Rhesus rotavirus stimulates 5-HT release from EC tumor cells in a dose-dependent manner. (A) Primary EC t.c. (n = 6) were infected with different concentrations of purified RRV and 5-HT secretion was measured in the cell culture supernatant at different time points by HPLC. (B) GOT1 and (C) primary EC t.c. were stimulated with RRV cell lysates (n = 3) (MOI = 1) and ultracentrifuged RRV cell lysates (n = 3) for 60 min and the 5-HT concentration was determined by ELISA. The asterisk (*) denotes statistical significance (P = 0.05; Mann Whitney test).

This observation is important since the release of 5-HT is known to be a Ca^2+^-dependent process in EC cells [35], including human enterochromaffin-like cells [36]. Using the cholera toxin (2 nM, 20 nM, 200 nM) as an agonist we observed a dose-dependent release of 5-HT from primary EC t.c. within 24 h (Figure. S4). RV was shown to stimulate the release of 5-HT in a dose and time-dependent manner with an increase of 5-HT release after 6 h p.i. in primary EC t.c. (Figure. 1A). Furthermore, when primary EC t.c. and GOT1 cells were incubated with supernatant from RRV-infected MA104 cells, a release of 5-HT was observed within 60 min (Figures. 1B, 1C). Such an early 5-HT response indicated that viral replication was unlikely to be required for 5-HT release. More likely, a release of viral protein(s) from infected cells could explain this effect. Purified double- and triple-shelled RV [37] (1 and 2 µg/ml, respectively) and virus particle-free supernatant (130000× g; 4 h, SW40) were individually added to GOT1 (500,000 cells/well) and primary EC t.c. (200,000 cells/well) for 60 min. While the ultracentrifuged supernatant stimulated 5-HT release within 60 min (Figures. 1B, 1C), no such effect was observed using the purified particles (data not shown). Therefore, it was concluded that double- and triple-shelled RV were not responsible for this early effect on 5-HT secretion.

### NSP4 stimulates release of 5-HT from GOT1 and primary enterochromaffin tumor cells

**Figure 2:**
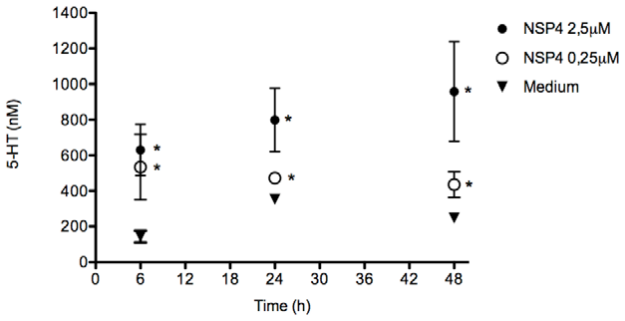
NSP4 induces 5-HT release in a dose- and time-dependent manner. Primary EC t.c. were incubated with recombinant NSP4 (n = 4) at different concentrations and the 5-HT concentration in the cell medium (n = 4) was determined at different time points by HPLC. The asterisk (*) denotes statistical significance (P<0.05; Mann Whitney test).

The enterotoxic glycoprotein NSP4 is secreted from human intestinal cells following RV infection [38], [39] and has been reported to mobilize intracellular Ca^2+^ from human HT-29 cells [40]. Therefore, we examined whether NSP4 secreted from polarized human intestinal Caco-2 cells infected with rotavirus [39] or recombinant NSP4 [41] could stimulate the release of 5-HT from GOT1 and primary EC t.c. Indeed NSP4 at concentrations ranging from 0.25–2.5 µM resulted in the secretion of 5-HT from primary EC t.c (Figure. 2). In a subsequent experiment, the addition of 2 µM NSP4 to GOT1 cells for as short time as 60 min revealed an increase of 204% of 5-HT in the media from cells stimulated with recombinant NSP4 (data not shown), and an increase of 84% of 5-HT from the secretory NSP4 (data not shown). Thus, both recombinant NSP4 and NSP4 secreted from rotavirus-infected Caco-2 cells were capable of stimulating the release of 5-HT from human EC t.c.

### NSP4 mobilizes intracellular Ca^2+^ in human enterochromaffin tumor cells

**Figure 3:**
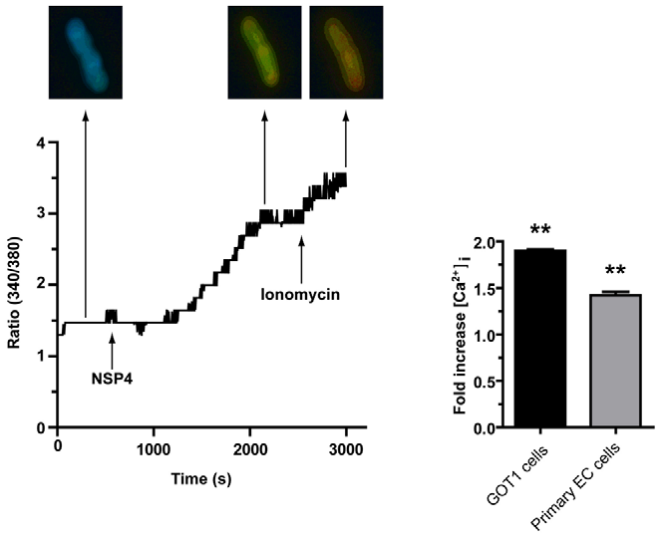
NSP4 induces an increase in intracellular Ca^2+^ in human EC tumor cells. GOT1 and primary EC t.c. were loaded with Fura-2 and Ca^2+^ measured before and after the addition of recombinant NSP4 (2 µM), for 40 min (GOT1 n = 8 cells, primary EC n = 10 cells). The asterisk (*) denotes statistical significance (P<0.01; Wilcoxon's rank sign test).

Both GOT1 and primary EC t.c. were loaded with the ratiometric fluorescent Ca^2+^ indicator Fura-2 and stimulated with NSP4. Following the establishment of a stable Fura-2 fluorescence baseline, recombinant NSP4 (2 µM) was added to cells and the Ca^2+^ signal measured continuously for 40–50 min. The intracellular Ca^2+^ concentration increased 1.5–2-fold in EC t.c. (GOT1 n = 8 cells, primary EC t.c. n = 10 cells) within 30 min of NSP4 stimulation (Figure. 3), which was consistent with observations in Caco-2 cells [34]. Ionomycin (1 µM) was added at the end of the experiment as a control to compare the magnitude of the increase, but it had little further effect, suggesting that NSP4 is a strong mobilizer of intracellular free Ca^2+^ (Figure. 3).

### NSP4 stimulates PLC-mediated inositol 1,4,5-trisphosphate production (IP_3_)

**Figure 4:**
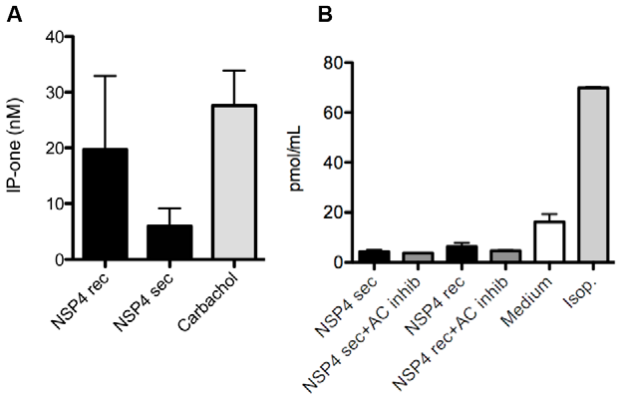
NSP4 activates phospholipase C mediated IP_3_ but not the cAMP pathway. (A) GOT1 cells were stimulated with secretory and recombinant NSP4 (2 µM) for 60 min during accumulation of IP-one. Carbachol were used as positive control. Supernatants were then analysed for the IP_3_ metabolite IP-one, using a commercial ELISA (n = 3). (B) GOT1 cells were stimulated with secretory or recombinant NSP4 (2 µM) for 5 min, with or without AC inhibitor, and cAMP was measured in cell lysates of stimulated cells (n = 3) by ELISA. Isoprotenerol (Isop.) were used as positive control.

Exogenous NSP4 can stimulate IP_3_ production in human intestinal HT-29 cells [40], and a human pancreatic carcinoid cell line (BON) was shown to produce IP_3_ after mechanical stimulation and activation of purinergic receptors [42]. We therefore determined weather NSP4 caused IP_3_ production in EC cells. The production of IP_3_ was indeed stimulated in GOT1 cells (400,000 cells/well) when incubated for 60 min with recombinant or secretory NSP4 (2 µM). Caracole (100 µM), a muscarinic receptor agonist known to activate the PLC pathway, was also used as an agonist. Both forms of NSP4 activated the PLC pathway, albeit to a less degree than carbachol (Figure. 4A).

Since activation of adenylyl cyclase (AC) and cyclic adenosine monophosphate (cAMP) signalling has been previously associated with Ca^2+^ increase and 5-HT release in GOT1 [43], KRJ-I [44] and BON cells [36], we next determined whether NSP4 could stimulate this pathway. GOT1 cells (650,000 cells/well) were thus stimulated with 2 µM NSP4 and the AC agonist isoproterenol (10 µm) for 5 and 30 min, followed by collection of the supernatant and quantification of cAMP as an indicator of AC activation. While isoproterenol stimulated cAMP in GOT1 cells, no effect of NSP4 was observed at 5 min (Figure. 4B) or 30 min (data not shown), suggesting that the cAMP pathway in GOT1 cells was not activated by NSP4.

### Rotavirus infects enterocytes in the close vicinity of enterochromaffin cells in the jejunum of mice

**Figure 5:**
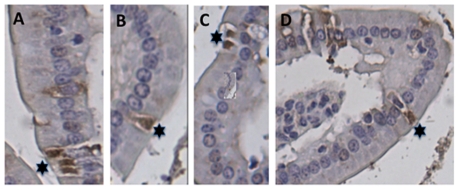
Localization of EC cells in the small intestine of mice. (A–D) Sections of paraffin-embedded small intestine tissue from uninfected BALB/c mice were processed for immunohistochemistry with rabbit anti-chromogranin A and peroxidase-labelled goat-anti rabbit. The EC cells were identified in all segments of the small intestine. (A) Ileum, (B–D) duodenum. Stars indicate chromogranin A containing-EC cells.

**Figure 6:**
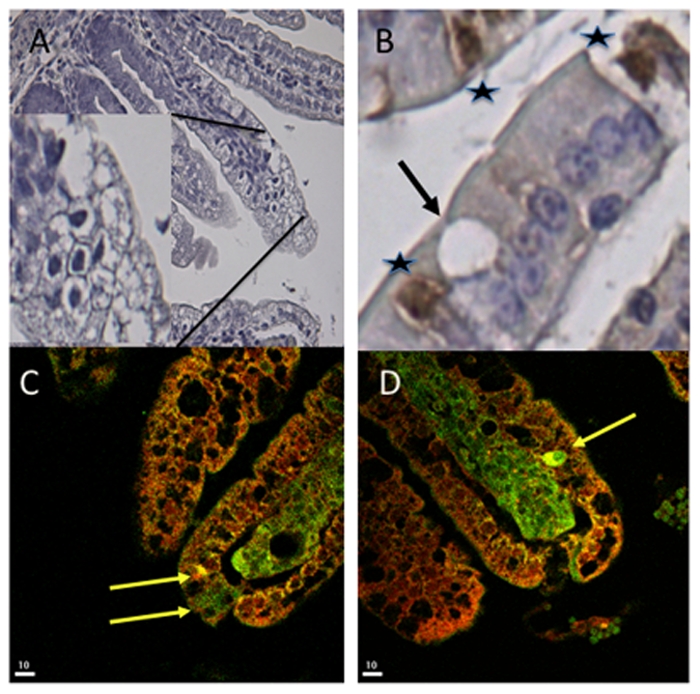
Co-localization of rotavirus and 5-HT in EC tumor cells. (A) Haematoxylin-stained jejunum of RV-infected mice. The picture shows extensive vacuoles characteristic of RV-infected enterocytes at 48 h p.i. (B) Sections of infected intestinal tissue were processed for immunohistochemistry for detection of EC cells with chromogranin A-specific antibodies. Stars indicate EC cells and the arrow identifies a vacuole in the close vicinity of an EC cell in the ileum. (C, D) Sections of jejunum intestinal tissue were stained for RV (red) and 5-HT (green). Arrows indicate EC cells with co-localization (yellow) of the virus and 5-HT.

The distribution and occurrence of neuroendocrine cells in the small intestine of the mouse duodenum, jejunum and ileum was assessed by immunohistochemistry for the neuroendocrine secretory granular marker chromogranin A. The duodenum, jejunum and ileum all contained neuroendocrine cells (Figures. 5A–D). They were observed in the crypts as well as among mature villous enterocytes, most abundantly in the duodenum. To investigate whether RV could infect enterocytes in the close vicinity of EC cells or infect chromogranin/5-HT-containing EC cells, infant mice were infected with murine rotavirus (strain EDIM) as described [5] and processed for histopathology and immunohistochemistry at different time points p.i. Figure 6A illustrates the typical vacuolization of infected mature enterocytes 48 h p.i. The vacuoles were occasionally found in the close vicinity of chromogranin-containing cells (Figure. 6B). No infected crypt cells were seen. Moreover, confocal microscopy revealed a co-localization between RV proteins and 5-HT-containing EC cells in jejunum of infected mice (Figures. 6C, 6D). Evidently, RV can infect enterocytes in the close vicinity of EC cells in the small intestine, and occasionally EC cells as well.

### 5-HT induces diarrhoea in infant mice

To further support our previous observations [3], that 5-HT participates in RV-induced illness, 50 µL 5-HT at 5 mg/kg (Serotonin creatinine sulfate monohydrate, Sigma Aldrich, Code: 85030) were administered intraperitoneally to five to seven days old Balb/C pups. Our objective was to investigate whether diarrhoea would occur. Following 30 min post administration, 5 of the 7 pups responded with diarrhoea, after 45 min 6 out of 7 and after 60 min all 7 responded with diarrhoea indistinguishable from RV–induced diarrhoea [3].

### Rotavirus induces neuronal activation in mice brain structures associated with vomiting

**Figure 7:**
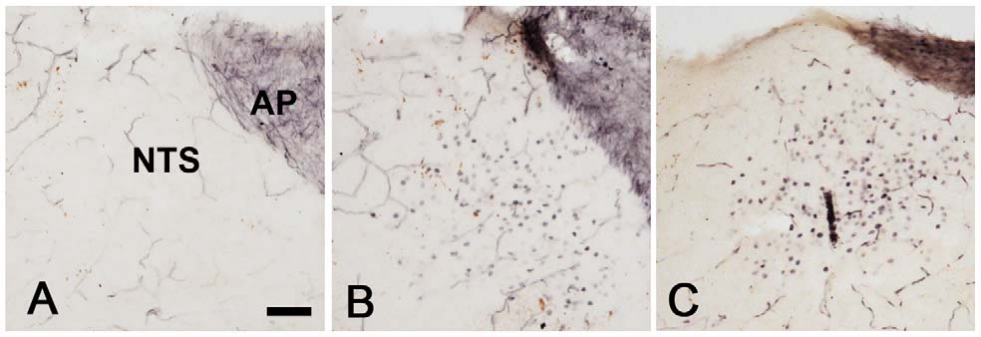
Brain activation in response to rotavirus infection. Coronal sections from the lower brainstem of a non-infected pup (A) and pups 48 h after RV infection (B, C). Note that many cells in the NTS of the infected animals display immunoreactivity for the activity marker Fos. AP; area postrema. Bar represents 50 µM.

To determine whether RV infection leads to activation of brain structures involved in sickness symptoms such as nausea and vomiting, immunohistochemical detection of Fos, a commonly used marker of neuronal activity was carried out on brain sections of RV-infected and uninfected mice [45]. Fos is an immediate early gene that is expressed upon repeated depolarization of neurons. In brains harvested 48 h. p.i., there was a robust activation of nucleus of the solitary tract, the main target structure for incoming fibers from the vagal nerve. This was seen in 3/5 of the infected animals. Thus, RV infection activated brain areas considered to be the vomiting centre. No activity was seen in uninfected animals (0/6) (Figure. 7). Our observation shows for the first time that RV activates brain structures associated with vomiting, presumably through activation of vagal afferents. No clear infection-induced Fos expression was seen in other parts of the brainstem.

## Discussion

Despite the clinical importance of vomiting induced by RV infection and its contribution to severe dehydration, no mechanism has yet been proposed for emesis. Progress in elucidating the RV-induced emetic mechanism has been hindered by lack of appropriate small animal models, because most commonly used animal models that are susceptible to RV, i.e. mice and rats, do not exhibit an emetic response [24]. Progress has been further restricted by the limited availability of human primary and established EC cell lines. Primary human carcinoid EC cells as well as the carcinoid EC cell line (GOT1), enable investigatigation of the role of EC cells in RV pathogenesis.

The EC cells have been identified from the mid- to the top of villi in the duodenum, jejunum and ileum, which are the segments associated with RV replication and histopathological lesions [46]. A cross-talk between EC cells and infected enterocytes is supported by immunohistochemistry and confocal microscopy experiments. The neuroendocrine cells were found in the close vicinity of cells with vacuoles, a characteristic feature of RV-infected enterocytes [46]–[48]. Moreover, RV sometimes co-localized with EC cells, suggesting a paracrine signalling of NSP4 and a cross-talk between EC cells and NSP4 *in vivo*.

Consistent with the proposal of EC cells being key cells in RV pathophysiology, we demonstrated that: (i) RV cause release of 5-HT from primary EC t.c., (ii) crude and virus particle-free supernatants from RV-infected MA104 cells stimulated 5-HT release within 60 min in primary and GOT1 EC t.c., (iii) recombinant and secretory NSP4 caused release of 5-HT from primary and GOT1 EC t.c. The latter finding is particularly interesting, and is to the best of our knowledge, the first observation of a virus/viral protein-mediated effect on human EC t.c., stimulating 5-HT release. This release exhibited similar time kinetics as the Ca^2+^ mobilization, which is consistent with previous findings in EC cells and BON cells [35], [36]. RV also stimulated 5-HT release from EC t.c. in a time and dose-dependent manner beginning about 6 h p.i. It is reasonable to believe that the 5-HT release and Ca^2+^ mobilization were associated with expression of NSP4.

Exogenous NSP4 can also cause the mobilization of intracellular Ca^2+^ [30], [31] via PLC-mediated IP_3_ production in human intestinal cells [40], [49]. Moreover, NSP4 is secreted from human intestinal cells [38] in a polarized fashion [39]. Therefore, we explored the novel hypothesis that RV, and particularly NSP4, stimulate EC t.c. via mobilization of intracellular Ca^2+^ and release of 5-HT. Since the elevation of Ca^2+^ after addition of NSP4 occurred after approx 20 min, similar to RV infections in Caco-2 cells [34], we speculate that the effect was not mediated by the opening of ion channels in the plasma membrane. It is more likely that it originated from internal ER stores as previously shown [31], although we cannot exclude that other possible mechanisms were involved. The kinetics of the Ca^2+^ response were similar to those of non-infected human intestinal Caco-2 cells inoculated with supernatant from RRV-infected Caco-2 cells at 18 h p.i. [34]. As the presence of proteins in the supernatant at this time point could not be explained by cell lysis, it was suggested that the Ca^2+^ mobilization is stimulated by viral proteins or peptides secreted from RV-infected cells [34]. Indeed, release of soluble NSP4 from RV-infected human intestinal cells has been demonstrated [38], [39], and we can now report that both recombinant NSP4 and NSP4 secreted from virus infected intestinal cells mobilize Ca^2+^ in EC t.c. and stimulate 5-HT release.

The EC cells express an ensemble of ligand-gated ion channels, chemo- and mechanosensitive-ion channels and G-protein-coupled receptors on their surface [50], [51]. G-protein-coupled AC and PLC are key enzymes involved in 5-HT release in EC cells [36], [44], [50], [52], [53]. Kolby and co-workers previously showed that GOT1 cells weakly responded to carbachol, a muscarinic receptor agonist and activator of PLC, suggesting that these cells lacked functional muscarinic receptors or had an impaired PLC-dependent formation of IP_3_ [43]. Consistent with this observation, we found only a modest response to carbachol and NSP4, indicating that NSP4 increases Ca^2+^ in an PLC-independent way [54].

In order to explain the mechanism, by which NSP4 alter the ER, it has been proposed that NSP4 stimulates Ca^2+^ signal transduction mechanisms by binding to specific surface membrane receptors, activates PLC [31], [40] and thus creating IP_3_. Suggested membrane receptors for NSP4, increasing intracellular Ca^2+^ are muscarinic receptors [31], [40] but also α1β1 and α2β1 integrins [55]. The mechanism by which NSP4 may alter ER is unknown. It has been hypothesized that NSP4 acts as a viroporin [54], forming a cation channel in the ER membrane or having a direct action on IP_3_ receptors in the ER membrane, with action or no action on the membrane itself [34]. Brunet and co-workers [34] reported that at a late stage of infection in Caco-2 cells, Ca^2+^ is partially increased by a PLC-dependent Ca^2+^ release from the ER through the opening of IP_3_-sensitive channels. However, they did not exclude an efflux of Ca^2+^ due to a direct alteration of the ER membrane.

GOT1 cells are believed to respond with an increase of intracellular Ca^2+^ concentration upon stimulation with the AC activator isoprotenerol [43]. We confirmed that isoprotenerol did indeed stimulate the formation of cAMP, but no such effect was observed with NSP4, suggesting that NSP4 does not induce the release of 5-HT through the AC pathway in these cells.

Activation of the vagal afferent fibres by toxins in the gut appears to operate via detection of toxins by EC cells, which release 5-HT to activate 5-HT_3_ receptors on vagal afferent fibres [56]. Induction of Fos has previously been observed in vomiting animals [57], [58] at the nucleus of the solitary tract (NTS) of CNS [58]. Our finding that RV-infected mice responded with a strong neural activation of the primary target site of vagal afferents, i.e. the NTS, is in line with the hypothesis that RV-induced activation of vagal afferents triggers vomiting. The fact that not all infected mice showed such activation (3/5) may be due to the time-kinetic variation of Fos activation between different animals. The peak period of Fos expression has shown to be 60–120 min after stimulation [59], and prolonged for up to 6 hours. Another study showed a long-term increase in Fos expression in the area postrema, the NTS and the nucleus amygdalae in conjunction with vomiting after cisplatin treatment in a animal species with an emetic response (the house musk shrew, *Suncus murinus*) [60]. They also showed that Fos activation in these brain stem areas could be suppressed by palonosetron, a 5-HT_3_ receptor antagonist, which is used as an anti-emetic drug. The time kinetics of RV-induced Fos expression needs to be further investigated, since the onset of clinical symptom (diarrhoea) in mice started around 24 h p.i and persisted, at least up to 96 h.

A limitation of using mice in these studies is the absence of a functional emetic reflex, but there are reports of “retching” but not vomiting, which may suggest that they have a degenerate reflex rather than none at all [56], [61]. Further support that 5-HT is associated with RV disease was provided by the observation that 5-HT induced diarrhoea in 7/7 animals within 60 min which is consistent with our previous observation that 5-HT_3_ receptor antagonists attenuate RV-induced diarrhoea [3]. The participation of EC cells in diarrhoea, as revealed by studies of 5-HT release and/or use of pharmacological blocking agents, has been demonstrated in such diverse fluid secretory states as those caused by cholera toxin [62], [63], the enterotoxins produced by *E coli* [64], bile salts [65] and *Salmonella typhimurium* [66]. Furthermore, an ENS involvement has been associated with cholera toxin [67], *E coli* heat stable toxin [68], certain bile salts [69], and gut inflammation [70]. These observations indicate that the interaction between EC cells and ENS is a pathophysiological mechanism common to many intestinal secretory states.

A *Staphylococcal* enterotoxin was recently reported to induce emesis by releasing 5-HT into the intestine, an effect inhibited by a 5-HT_3_ receptor antagonist [18]. Similarly, our data show that the NSP4 enterotoxin stimulated release of 5-HT and, sometimes, RV was localized to EC cells in the small intestine. Support for our hypothesis that RV-induced vomiting includes stimulation of EC cells, 5-HT and activation of vagal afferents is derived from several clinical studies with 5-HT_3_ receptor antagonists. For example, ondansetron, a 5-HT_3_ receptor antagonists, has successfully been used to attenuate vomiting in paediatric gastroenteritis [20], [21], [23], [71], [72]. Furthermore, it has also been reported that ondansetron-treatment of vomiting in American children has become quite common [21]. While it is established that vomiting during acute gastroenteritis in young children can be treated with 5-HT_3_ receptor antagonists, it remains to be shown in clinical studies that children with RV-induced vomiting can be successfully treated. Moreover, the emetic responses to the *Staphylococcus* toxin in the musk shrew animal model [18], suggests that this animal model might be explored to study RV–induced vomiting.

**Figure 8:**
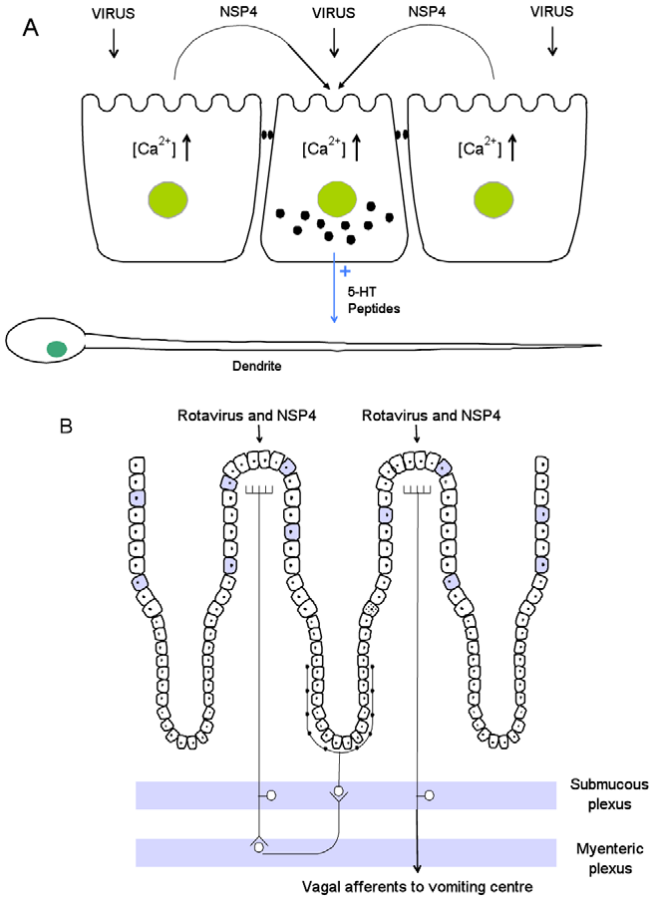
Proposed disease mechanisms for rotavirus infection. Panel A. Interplay between RV, NSP4, EC cells and enteric nervous dendrites. RV infects mature enterocytes evoking the release of NSP4, which together with invading virus, causes the release of 5-HT from EC cells. This, in turn, stimulates nervous dendrites located adjacent to the EC cells. *Panel B*. Physiological effects of 5-HT release from EC cells. It seems possible that the released 5-HT may activate both intrinsic afferents of the ENS as well as extrinsic afferent nerves. It is proposed that the stimulated intrinsic afferents constitute part of a nervous secretory reflex within ENS (left part of panel B), causing an intestinal fluid loss. Stimulation of extrinsic vagal afferents, activates NTS, a brain structure associated with nausea and vomiting (right part of panel B). The picture depicts the simplest nervous, secretory model that can be constructed on the basis of current experimental observations. Filled cells represent EC cells.

Our present and previous studies of the pathophysiology of RV infections [2], [3] suggest a common triggering mechanism for the fluid loss and the emesis as schematically illustrated on Figure 8. The results of the present study strongly suggest that RV per se and/or NSP4 released from adjacent virus-infected enterocytes increase intracellular Ca^2+^concentration in the EC cells, which, in turn, stimulates the release of 5-HT from EC cells. We propose that the released 5-HT activates both intrinsic and extrinsic afferent nerve fibres located in close vicinity to the EC cells. Hence, EC cells function as sensory transducers of different luminal stimuli. Such a mechanism has been demonstrated in several experimental models [8], [9], [42]. As pointed out above intrinsic afferent nerves stimulated by the released 5-HT are part of intramural nervous reflex(es), which in the case of RV infection increase fluid secretion from intestinal crypts via the release of VIP (vasoactive intestinal peptide) at the crypt epithelium [3], [4]. The released 5-HT also activates vagal afferents that project to the medullary vomiting centre of the central nervous system, triggering the emetic reflex [25], [26], [42], [50]. It is apparent that EC cells and nerves play an important role for RV-induced diarrhoea and vomiting and the present findings may be of more general importance for our understanding of pathophysiological mechanisms of many different types of infection-induced diarrhoea and vomiting.

Our observations may be of clinical importance, since the possibility to reduce vomiting by 5-HT_3_ receptor antagonists in acute viral gastroenteritis will both favour oral rehydration therapy by preventing vomiting and attenuate fluid loss, thus reducing hospitalisation of children [21].

## Materials and Methods

### Cell culture

Primary tumor EC cells [43] and a human midgut carcinoid tumor cell line (GOT1), previously characterized for specific EC cell markers [43], were cultured in RPMI 1640 medium supplemented with 10% FCS, 0.73 mg/ml L-glutamine, 5 µg/ml apo-transferrin, 5 µg/ml insulin, and PEST (100 U penicillin, 100 µg/ml Streptomycin). Primary EC cells were obtained from patients with liver metastasis and midgut carcinoid syndrome [43].

### Rotavirus infection and quantification

RRV was cultivated, quantified and purified as described [37], [73]. The GOT1 cells (200,000/well) were infected with trypsin-activated RRV at an MOI of 0.1 as previously described [73]. Briefly, after infection the cells were washed twice and then incubated with Minimal Essential Medium (MEM) containing trypsin (T8353, bovine pancreas, type III, Sigma Aldrich), 1 µg/ml medium, for 3, 24 and 48 h. At each time point cells and supernatants were collected and frozen at −80°C. Cell suspensions were freeze-thawed twice, centrifuged to remove cell debris, followed by determination of the progeny virus, as previously described [74].

### Fura-2/AM loading and measurement of intracellular Ca^2+^

The GOT1 cells (450,000 cells/plate) and primary EC t.c. (200,000 cells/plate) were seeded onto coverslip-bottomed plastic Petri dishes used for fluorescent microscopy (MatTek Corporation). The cells were washed twice with MEM without FCS and then loaded with the fluorescent Ca^2+^ indicator Fura-2-AM (Molecular Probes), 10 µM in the presence of Pluronic-F 127 (Sigma Aldrich), 20% w/v in DMSO, 10 µl/plate in a total volume of 1 ml MEM without FCS for 45 min at 37°C. The cells were washed twice with MEM without FCS and incubated in fresh medium. Ratiometric imaging of Fura-2-loaded cells was performed using a Photon Technology International (Monmouth Junction, NJ) system and a Zeiss Axiovert 100 M (Jena, Germany) microscope equipped with a ×100 glycerol-immersion Fluar objective (1.3 numerical aperture) and a PTI IC-200 camera for fluorescence imaging. Before adding NSP4 to the cell cultures, initial 10 min fluorescence (F340/F380) was captured to obtain a Ca^2+^-baseline. Bright-field images were taken simultaneously using a PTI IC-100 camera by passing the transmission light through a 700 nm band-pass filter in front of the halogen lamp to avoid stray light in the fluorescence channel. NSP4 were added to a final concentration of 125 nM and 2 µM, respectively, and continuously measured in real time for further 30 min. For measuring of intracellular Ca^2+^ using RRV see supporting information (Protocol. S1 and S2).

### Immunofluorescence

The GOT1 and primary EC-cells were stained for 5-HT and chromogranin A. Cells were fixed with 4% paraformaldehyde/PBS on microscope slides (Histolab, Göteborg, Sweden) overnight at 4°C in a humidity chamber and then processed for immunofluorescence (Protocol. S3).

### Purification of recombinant and secreted NSP4

Histidine-tagged NSP4 from a simian rotavirus strain (SA11) was produced using the baculovirus expression vector system and *Spodoptera frugiperda* (Sf9) insect cells. The NSP4 was purified by column chromatography, as previously described [41]. Secreted NSP4 was purified from the media of polarized epithelial Caco-2 cells infected with bovine rotavirus (UK strain) [39]. Briefly, the medium was ultracentrifuged to remove virions and NSP4 purified by sequential concanavalin A affinity chromatography and monoS cation exchange chromatography. The protein was judged as >99% pure by SDS PAGE and silver staining.

### Cholera toxin

The cholera toxin was purchased from List Biological Laboratories, Campbell, California; Code 101A.

### Detection of 5-HT

The 5-HT in cell culture medium from primary EC t.c. and GOT1 cells was quantified using a commercial serotonin ELISA kit (IBL International, Hamburg, Germany; Code: RE59121) according to the manufacturer's instructions, or by HPLC [43].

### cAMP assay

GOT1 cells (650,000/well) were pre-incubated for 30 min in MEM without FCS with (100 µM) or without the AC inhibitor 2′, 5′-Dideoxyadenosine, (Sigma Aldrich; Code: D7408), at 37°C with 5% CO_2_. Recombinant or secretory NSP4 (2 µM), were added for 5 and 30 min. Cells were lysed and analysed for intracellular cAMP with a commercial cAMP EIA kit as described by the manufacture (R&D systems, United Kingdom; Cat.No KGE002B). MEM without FCS was used as a negative control and Isoprotenerol (10 µM; Sigma Aldrich; Code:I6504) as a positive control.

### IP-one assay

IP_3_ production was measured indirectly through the accumulation and analysis of the metabolite IP-one. GOT1 cells (650,000/well) were stimulated with recombinant NSP4 or secretory NSP4, 2 µM, for 60 min at 37°C with 5% CO_2_ in a buffer containing LiCl (50 mM) to prevent degradation of IP-one. Carbachol (Sigma Aldrich, USA), 100 µM, were used as positive control. Cells were lysed and analysed using a commercial IP-one ELISA kit (Cisbio Bioassay, France; Code: 72IP1PEA), according to the manufacturer's instructions.

### Mice

RV naïve, five to seven days old BALB/c mice (B&K Laboratories, Sollentuna, Sweden) were orally infected with 10 µL/animal (100DD_50_ diarrhoea doses) of murine rotavirus strain EDIM [3], [46]. For mice receiving 5-HT (Sigma Aldrich), 50 uL at 5 mg/kg were administered intraperionally and observed for signs of diarrhoea at 30, 45 and 60 minutes after administration. The small intestines were removed and processed as previously described [3], [46].

### Immunohistochemistry of enterochromaffin cells

For immunohistochemistry of mice intestinal tissue, paraffin-embedded specimens were cut into thin sections as previously described [46]. Intestinal sections were hydrated and processed for immunohistochemistry (Protocol. S4).

### Confocal microscopy of rotavirus-infected intestinal EC cells

For immunoflourescence staining, the paraffin-embedded intestinal specimens were cut into thin sections, hydrated and processed for immunohistochemistry (Protocol. S5).

### Fos-staining

Brains were cut at a freezing microtome at 30 micrometer and further processed for free floating immunohistochemistry. Sections were incubated with a primary antibody directed against Fos (Santa Cruz Biotechnology; sc-52; 1∶1000) and the antibody was visualized using avidin-biotin complex amplification and DAB as chromogen according to previously published protocols [75].

### Statistical analysis

The results are expressed as mean ± standard errors of the mean (SEM), unless indicated. Statistical analysis of all data was performed using the repeated measures analysis. The Mann Whitney test was used unless stated. P-value of ≤0.05 was considered significant.

### Ethics statement

All animal experiments in this study were carried out in strict accordance with the recommendations in the guide for the care and use of laboratory animals conformed to Swedish animal protection laws and applicable guidelines (djurskyddslagen 1988:534; djurskyddsförordningen 1988:539; djurskyddsmyndigheten DFS 2004:4). Animal experiments were approved by the local Ethical Committee (Stockholm Norra Djurförsöksetiska nämnd, Stockholm, Sweden; Approval No: N289/09). All efforts were made to minimize suffering and surgical operations were performed afterwards the animals were euthanized by overdose of Isoflurane.

## Supporting Information

Figure S1 **Identification of chromogranin A and 5-HT in EC tumor cells.** Primary EC t.c. and GOT1 cells were incubated with anti-chromogranin A (rhodamine) and 5-HT (FITC) specific antibodies. The DNA staining with 4′,6-diamidino-2-phenylindole (DAPI) shows that all GOT1 cells and more than 95% of the primary cells were positive for chromogranin A and 68% of the GOT1 cells and 40% of the primary cells were positive for 5-HT.
(TIF)

Figure S2 **Rotavirus replicates in EC tumor cells.** Primary EC t.c. were infected with RRV and examined by double immunofluorescence at 14 h p.i. Fluorescence shows RV (A), Chromogranin A (B) and merged pictures (C).
(TIF)

Figure S3 **Supernatant from rotavirus infected cell lysates induces an increase in intracellular free Ca^2+^.** GOT1 and primary EC t.c. were loaded with Fura-2 and Ca^2+^ measured before and after infection with RRV, as described in protocol S1 and S2. (A) Before infection and (B) 1 h p.i. (C) Relative increases in % of intracellular free Ca^2^ at 1 hr p.i. in GOT1 and primary EC cells. For either cell type 9 and 7 regions of interest (ROI) were assessed before infection, and 8 and 9 ROIs after infection, respectively. Since each ROI can contain two or more adjacent cells, at least 14 cells were measured in each case. The Ca^2+^ concentrations, as described by the F340/F380 ratios, were before and after 1 h p.i., 1,76 and 2,75 for GOT1 cells, and 1,45 and 2,43 for primary EC cells, respectively. These values corresponds roughly to Ca^2+^ concentrations of 300–400 and 1100–1200 nM, and 200 and 1000 nM for the GOT1 and primary EC cells, respectively [76].
(TIF)

Figure S4 **Cholera toxin stimulates 5-HT release from EC tumor cells in dose-dependent manner.** Primary EC t.c. were stimulated with different concentrations of cholera toxin for 24 hours followed by determination of 5-HT with HPLC (n = 5). The asterisk (*) denotes statistical significance (P<0,05; Mann Whitney test).
(TIF)

Protocol S1 **Supporting method file of Fura-2/AM loading for calcium measurements.**
(DOC)

Protocol S2 **Supporting method file for Ca^2+^ imaging of rotavirus infected enterochromaffin cells.**
(DOC)

Protocol S3 **Supporting method file for immunofluorescence staining of** ***in vitro*** **enterochromaffin cells.**
(DOC)

Protocol S4 **Supporting method file for immunohistochemistry of intestinal enterochromaffin cells of mice.**
(DOC)

Protocol S5 **Supporting method file for confocal microscopy of rotavirus-infected intestinal EC cells.**
(DOC)
