# Supplementary figures and images for: Rotavirus Stimulates Release of Serotonin (5-HT) from Human Enterochromaffin Cells and Activates Brain Structures Involved in Nausea and Vomiting
Source: PLoS Pathog. 2011 Jul 14;7(7):e1002115. doi: 10.1371/journal.ppat.1002115 (PMC3136449; doi:10.1371/journal.ppat.1002115)

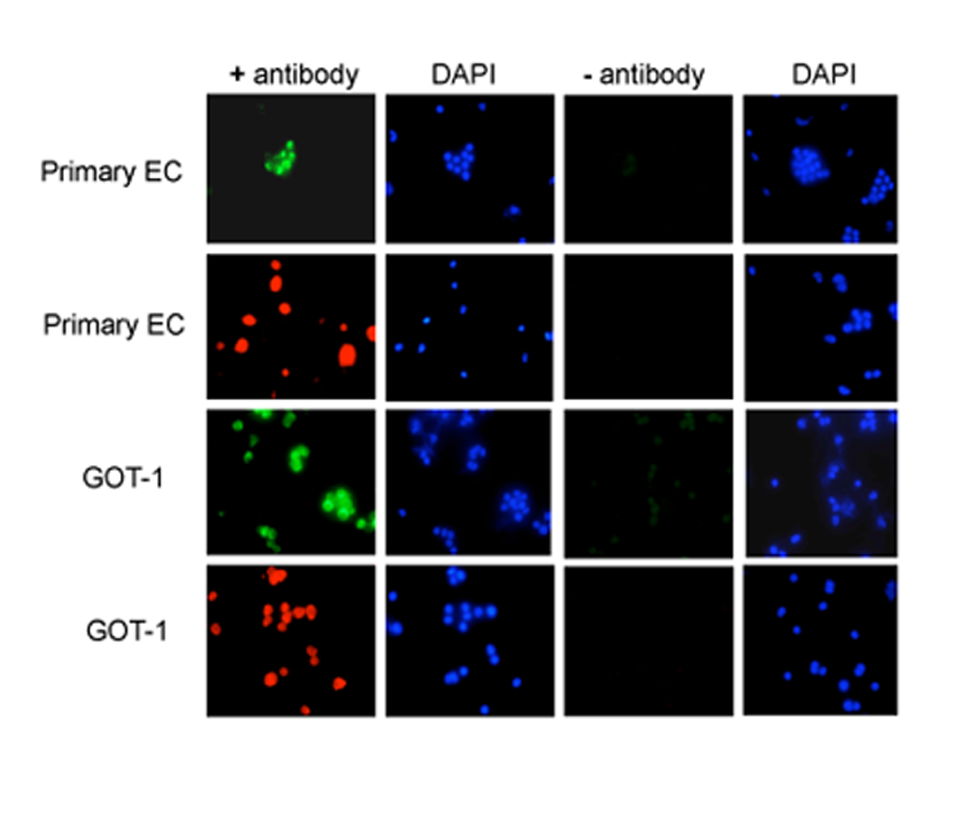

Supplement: Figure S1 — Identification of chromogranin A and 5-HT in EC tumor cells. Primary EC t.c. and GOT1 cells were incubated with anti-chromogranin A (rhodamine) and 5-HT (FITC) specific antibodies. The DNA staining with 4′,6-diamidino-2-phenylindole (DAPI) shows that all GOT1 cells and more than 95% of the primary cells were positive for chromogranin A and 68% of the GOT1 cells and 40% of the primary cells were positive for 5-HT. (TIF) [file ppat.1002115.s001.tif]

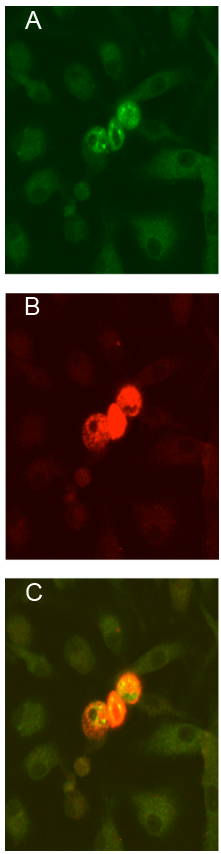

Supplement: Figure S2 — Rotavirus replicates in EC tumor cells. Primary EC t.c. were infected with RRV and examined by double immunofluorescence at 14 h p.i. Fluorescence shows RV (A), Chromogranin A (B) and merged pictures (C). (TIF) [file ppat.1002115.s002.tif]

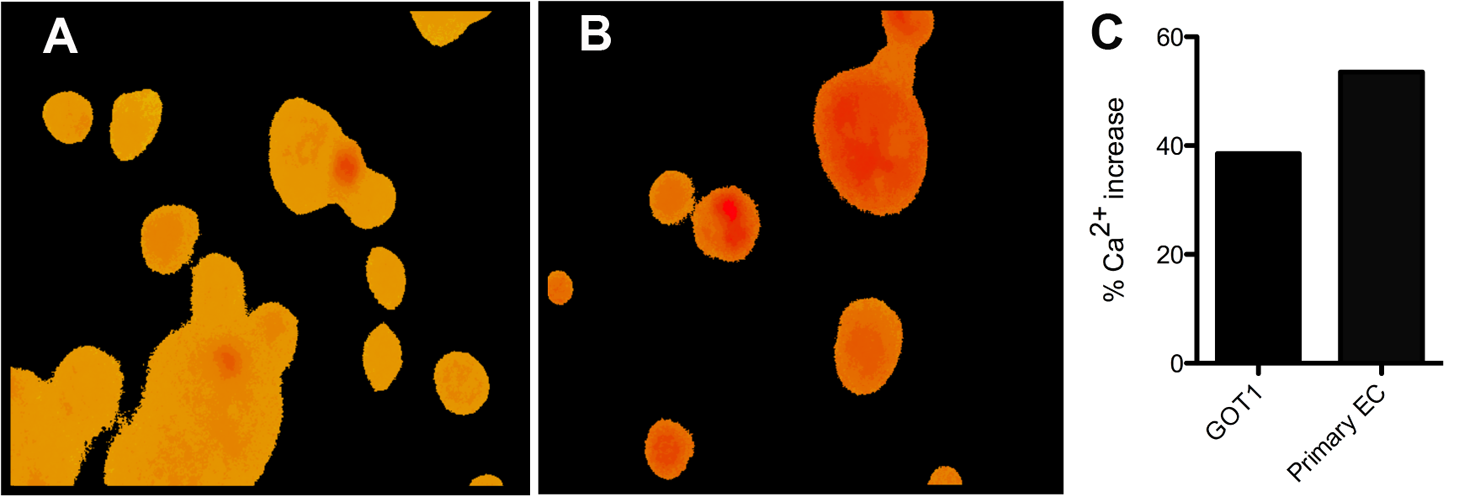

Supplement: Figure S3 — Supernatant from rotavirus infected cell lysates induces an increase in intracellular free Ca2+. GOT1 and primary EC t.c. were loaded with Fura-2 and Ca2+ measured before and after infection with RRV, as described in protocol S1 and S2. (A) Before infection and (B) 1 h p.i. (C) Relative increases in % of intracellular free Ca2 at 1 hr p.i. in GOT1 and primary EC cells. For either cell type 9 and 7 regions of interest (ROI) were assessed before infection, and 8 and 9 ROIs after infection, respectively. Since each ROI can contain two or more adjacent cells, at least 14 cells were measured in each case. The Ca2+ concentrations, as described by the F340/F380 ratios, were before and after 1 h p.i., 1,76 and 2,75 for GOT1 cells, and 1,45 and 2,43 for primary EC cells, respectively. These values corresponds roughly to Ca2+ concentrations of 300–400 and 1100–1200 nM, and 200 and 1000 nM for the GOT1 and primary EC cells, respectively [76]. (TIF) [file ppat.1002115.s003.tif]

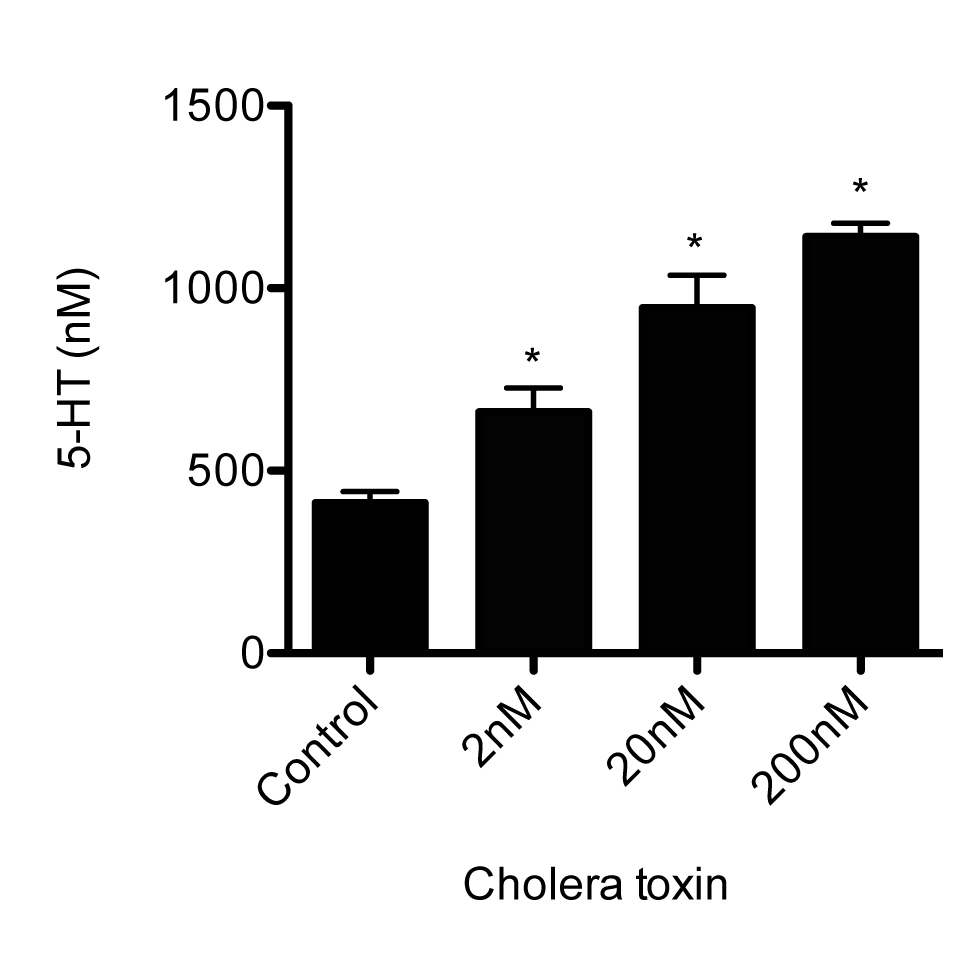

Supplement: Figure S4 — Cholera toxin stimulates 5-HT release from EC tumor cells in dose-dependent manner. Primary EC t.c. were stimulated with different concentrations of cholera toxin for 24 hours followed by determination of 5-HT with HPLC (n = 5). The asterisk (*) denotes statistical significance (P<0,05; Mann Whitney test). (TIF) [file ppat.1002115.s004.tif]
