# Supplementary material for: Rotavirus Stimulates Release of Serotonin (5-HT) from Human Enterochromaffin Cells and Activates Brain Structures Involved in Nausea and Vomiting
Source: PLoS Pathog. 2011 Jul 14;7(7):e1002115. doi: 10.1371/journal.ppat.1002115 (PMC3136449; doi:10.1371/journal.ppat.1002115)
Supplement: Protocol S1 — Supporting method file of Fura-2/AM loading for calcium measurements. (DOC) [file ppat.1002115.s005.doc]

**Protocol S1.**

**Fura-2/AM Loading for calcium measurements.**

GOT1 (450.000 cells/plate) and primary EC t.c. (200.000 cells/plate) were plated on plastic petridishes used for fluorescent microscopy (MatTek Corporation, MA, USA) and incubated for 2 days at 37°C and 5% CO_2_ in RPMI media. Cells were then washed twice with Krebs–Ringer Glucose (KRG) buffer with Ca^2+^ (1mM) or without Ca^2+^ (Linköping University Hospital, Sweden) and then loaded with the fluorescent Ca^2+^ indicator 1[2-(5-carboxyoxazol-2-yl)-6-aminobenzoFuran-5-oxyl]-2-(2’-amino-5-methylphenoxy)-ethane-N,N,N,N’,N’-tetraacetic acid (Fura-2-AM), 10μM (Molecular Probes, Inc., Eugene, OR), Pluronic-F 127, (20% w/v in DMSO) 10μl/plate (Sigma Aldrich, St Louis, MO) and Probenecid, 5μM (Sigma Aldrich) in 1ml KRG buffer with and without Ca^2+^ and incubated for 45 min at 37°C. Cells were then washed 2 times with KRG buffer with and without Ca^2+^ and then incubated with KRG with and without Ca^2+^ for 40 min at 37°C and 5% CO_2_.
