# Supplementary material for: Rotavirus Stimulates Release of Serotonin (5-HT) from Human Enterochromaffin Cells and Activates Brain Structures Involved in Nausea and Vomiting
Source: PLoS Pathog. 2011 Jul 14;7(7):e1002115. doi: 10.1371/journal.ppat.1002115 (PMC3136449; doi:10.1371/journal.ppat.1002115)
Supplement: Protocol S2 — Supporting method file for Ca2+ imaging of rotavirus infected enterochromaffin cells. (DOC) [file ppat.1002115.s006.doc]

**Protocol S2.**

**Ca^2+^ imaging of rotavirus infected enterochromaffin cells.**

Ratio imaging of Fura-2-loaded cells was done with a Photon Technology International (Monmouth Junction, NJ) system and a Zeiss Axiovert 100 M (Jena, Germany) microscope equipped with a 40x or 100x glycerol-immersion Fluar objectives (1.3 numerical aperture). Before infecting the cell cultures with virus, an initial 10 image pairs (F340/F380) for approximately 2 min, were captured to obtain a Ca^2+^-baseline. The cells were infected with trypsin-activated rhesus rotavirus (RRV), GOT1 cells at an multiplicity of infection (MOI) of 3 and primary tumor cells at an MOI of 10 in Eagles MEM (Gibco, Invitrogen Paisley, UK). The virus was incubated with the cells for 60 min and Ca^2+^-imaging was then repeated as described above at 1, 5, and 23 hr p.i. Images were taken every 6 sec and represent the mean of 8 successive video frames. Bright-field images were captured simultaneously using a Newvicon video-camera (Hamamatsu C-2400, Hamamatsu Photonics K.K, Hamamatsu City, Japan) by passing the transmission light through a 700-nm band-pass filter in front of the halogen lamp to avoid stray light in the fluorescence channel. The Fura-2 experiments were performed in KRG buffer with Ca^2+^ (1mM) and without Ca^2+^ (Linköping University Hospital, Sweden).
