# Supplementary material for: Rotavirus Stimulates Release of Serotonin (5-HT) from Human Enterochromaffin Cells and Activates Brain Structures Involved in Nausea and Vomiting
Source: PLoS Pathog. 2011 Jul 14;7(7):e1002115. doi: 10.1371/journal.ppat.1002115 (PMC3136449; doi:10.1371/journal.ppat.1002115)
Supplement: Protocol S3 — Supporting method file for immunofluorescence staining of in vitro enterochromaffin cells. (DOC) [file ppat.1002115.s007.doc]

**Protocol S3.**

**Immunofluorescence staining of *in vitro* enterochromaffin cells.**

The GOT1 and primary EC-cells were stained for 5-HT and chromogranin A. Cells were fixed with 4% paraformaldehyde/PBS on microscope slides (Histolab, Göteborg, Sweden) overnight at 4 °C in a humidity chamber and then processed for immunofluorescence as follows. Briefly, they were washed twice with PBS, permeabilized with 1% Tween (Sigma Aldrich) in PBS for 10 min, washed once with PBS and then blocked with 1% bovine serum albumin (BSA) (Sigma Aldrich) in PBS, followed by rabbit anti-chromogranin A antibody (Bachem, Bubendorf, Switzerland; Code: T-4485.0050), diluted 1:50 in PBS, or mouse anti-serotonin antibody (Dako Cytomation, Glostrup, Denmark; Code: M758), diluted 1:50 in PBS, for 30 min at RT in a humidity chamber. Following washes (×3) with PBS, rhodamine goat anti-rabbit IgG (Jackson Immuno Research, West Grove, PA, USA), diluted 1:400 in PBS, or fluorescein (FITC) goat anti-mouse IgG (Jackson Immuno Research), diluted 1:200 in PBS, was added for 60 min at RT in a humidity chamber. Nuclei were counterstained with 4',6'-diamidino-2-phenylindole (DAPI) (Invitrogen, Paisley, UK; Code: D1306) of concentration 300 nM in PBS for 5 min and then washed (×3) with PBS. We mounted the slides with mounting medium (DAKO Cytomation, Copenhagen, Denmark; Code: S3023), examined them using fluorescence microscopy (Nikon eclipse E600) and captured photos using a digital camera (Nikon digital camera DXM 1200F, Tokyo, Japan).
