# Supplementary material for: Rotavirus Stimulates Release of Serotonin (5-HT) from Human Enterochromaffin Cells and Activates Brain Structures Involved in Nausea and Vomiting
Source: PLoS Pathog. 2011 Jul 14;7(7):e1002115. doi: 10.1371/journal.ppat.1002115 (PMC3136449; doi:10.1371/journal.ppat.1002115)
Supplement: Protocol S4 — Supporting method file for immunohistochemistry of intestinal enterochromaffin cells of mice. (DOC) [file ppat.1002115.s008.doc]

**Protocol S4.**

**Immunohistochemistry of intestinal enterochromaffin cells of mice.**

For immunohistochemistry, paraffin-embedded specimens were cut into thin sections as previously described [46]. Briefly, intestinal segments were hydrated for 5 min in Xylen (×2) (Histolab, Goteborg, Sweden), followed by 100% ethanol (×2), 95% ethanol (×1), 70% ethanol (×1) and finally in washing buffer (0.05 M Tris-HCL/0.9% NaCl, pH 7.6). The slides were then incubated for 10 min in washing buffer containing 30% H_2_O_2_ to remove endogenous peroxidase followed by washes (×3) with washing buffer. To block non-specific reactions the intestinal segments were incubated in washing buffer with 1% BSA for 30 min at RT in a humid chamber.

A rabbit anti-chromogranin A antibody (Peninsula Lab Inc., Bachem Group, CA, USA), diluted 1:50 in washing buffer, was added to samples for 60 min at 37 ºC in a humid chamber. The slides were washed with washing buffer (×3) and HRP-conjugated goat anti- rabbit IgG (Biorad, Sweden), diluted 1:1000 in washing buffer, was added to the slides followed by incubation at 37 °C for 60 min in a humid chamber.

After washing the slides with washing buffer (×3), DAB staining substrate (Saveen Werner AB, Stockholm, Sweden) (1 ml DAB solution + 50 ml washing buffer +10 μl H2O2 (30%) was added for 3-5 min followed washes (×3) in distilled water. Finally, the slides were stained with haematoxylin for 4 seconds then washed under running water for a few minutes. Samples were dehydrated by passing specimens through the following solutions: 70% ethanol (×1), 95% ethanol (×1), 100% ethanol (×2), Xylen (×2). After the last bath Pertex liquid oil (Histolab, Göteborg, Sweden) was added and the specimens were dried at RT in order to be examined under the microscope.
