# Supplementary material for: Rotavirus Stimulates Release of Serotonin (5-HT) from Human Enterochromaffin Cells and Activates Brain Structures Involved in Nausea and Vomiting
Source: PLoS Pathog. 2011 Jul 14;7(7):e1002115. doi: 10.1371/journal.ppat.1002115 (PMC3136449; doi:10.1371/journal.ppat.1002115)
Supplement: Protocol S5 — Supporting method file for confocal microscopy of rotavirus-infected intestinal EC cells. (DOC) [file ppat.1002115.s009.doc]

**Protocol S5.**

**Confocal microscopy of rotavirus-infected intestinal EC cells.**

For immunoflourescence staining, the paraffin-embedded intestinal specimens were cut into thin sections and the tissues were hydrated as for immunohistochemistry. Specimens were washed (×3) with washing buffer (0.05 M Tris-HCL/0.9% NaCl, pH 7.6) and incubated with BSA 1% in washing buffer for 30 min at RT in a humid chamber. After removing the BSA two antibody mixtures were added. First mixture: a mouse RV anti-vp6 monoclonal antibody (Mab255, kindly provided by Harry Greenberg), diluted 1:50 in washing buffer, mixed with rabbit anti-chromogranin A antibody (Peninsula Lab Inc., Bachem Group, CA), diluted 1:50 in washing buffer. Second mixture: a rabbit polyclonal RV antiserum (K224 SBL, Stockholm, Sweden), diluted 1:50 in washing buffer, was mixed with mouse anti-serotonin antibody (Dako Cytomation, Glasdrup, Denmark), diluted 1:50 in washing buffer. After 60 min incubation at 37 °C in a humid chamber the slides were washed (×3) with washing buffer and incubated with FITC-conjugated goat anti-mouse IgG (Jacksson Immuno Research), and rhodamine-conjugated goat anti-rabbit IgG (Jacksson Immuno Research, USA), both antibodies diluted 1:50 in washing buffer. Following 60 min incubation at 37 °C in a humid chamber and washing (×3) with washing buffer, the specimens were mounted (DAKO Fluorescent Mounting Medium) and examined using a confocal microscope (Bio-Rad Radiance 2100-MP, Bio-Rad-CarlZeiss, Jena, Germany).
